# Independent Evolution of Winner Traits without Whole Genome Duplication in *Dekkera* Yeasts

**DOI:** 10.1371/journal.pone.0155140

**Published:** 2016-05-06

**Authors:** Yi-Cheng Guo, Lin Zhang, Shao-Xing Dai, Wen-Xing Li, Jun-Juan Zheng, Gong-Hua Li, Jing-Fei Huang

**Affiliations:** 1 School of Life Sciences, University of Science and Technology of China, Hefei, Anhui, P.R. China; 2 State Key Laboratory of Genetic Resources and Evolution, Kunming Institute of Zoology, Chinese Academy of Sciences, Kunming, Yunnan, China; 3 Graduate School of Life Science, Tohoku University, Sendai, Miyagi, Japan; 4 Institute of Health Sciences, Anhui University, Hefei, Anhui, China; Fred Hutchinson Cancer Research Center, UNITED STATES

## Abstract

*Dekkera* yeasts have often been considered as alternative sources of ethanol production that could compete with *S*. *cerevisiae*. The two lineages of yeasts independently evolved traits that include high glucose and ethanol tolerance, aerobic fermentation, and a rapid ethanol fermentation rate. The *Saccharomyces* yeasts attained these traits mainly through whole genome duplication approximately 100 million years ago (Mya). However, the *Dekkera* yeasts, which were separated from *S*. *cerevisiae* approximately 200 Mya, did not undergo whole genome duplication (WGD) but still occupy a niche similar to *S*. *cerevisiae*. Upon analysis of two *Dekkera* yeasts and five closely related non-WGD yeasts, we found that a massive loss of *cis*-regulatory elements occurred in an ancestor of the *Dekkera* yeasts, which led to improved mitochondrial functions similar to the *S*. *cerevisiae* yeasts. The evolutionary analysis indicated that genes involved in the transcription and translation process exhibited faster evolution in the *Dekkera* yeasts. We detected 90 positively selected genes, suggesting that the *Dekkera* yeasts evolved an efficient translation system to facilitate adaptive evolution. Moreover, we identified that 12 vacuolar H^+^-ATPase (V-ATPase) function genes that were under positive selection, which assists in developing tolerance to high alcohol and high sugar stress. We also revealed that the enzyme *PGK1* is responsible for the increased rate of glycolysis in the *Dekkera* yeasts. These results provide important insights to understand the independent adaptive evolution of the *Dekkera* yeasts and provide tools for genetic modification promoting industrial usage.

## Introduction

Yeasts usually win the competition in sugar rich environment and become the predominant group in nature [1]. The baker's yeast *Saccharomyces cerevisiae* was the most representative winner which could rapidly convert sugars into ethanol even under aerobic conditions. This aerobic fermentation is also known as the Crabtree effect [2]. When sugars are abundant in the environment, *S*. *cerevisiae* transforms sugars into ethanol, which results in the inhibition of other microorganisms. *S*. *cerevisiae* also uses ethanol as a carbon source to produce energy and growth. This "make–accumulate–consume" lifestyle has benefited *S*. *cerevisiae* and its close relatives throughout history [3, 4]. Fast sugar consumption, ethanol production, accumulation and tolerance, and the ability to propagate without oxygen are the traits responsible for *S*. *cerevisiae* becoming the "winner" in nature and industrial ethanol production [5]. However, nonconventional yeast species, *Dekkera* yeasts, have been observed to compete with *S*. *cerevisiae* in several ethanol production plants that contain low concentrations of sugars [6].

*Dekkera* yeasts are a major cause of wine spoilage worldwide [7] due to the production of volatile by-products. It has been estimated that *D*. *bruxellensis* diverged from a common ancestor of *S*. *cerevisiae* 200 million years ago [5]. However, these two phylogenetically distant yeasts have independently evolved similar features beneficial for ethanol production. They are also often found in the same habitats and share several food-related traits, such as the production of higher levels of ethanol and the ability to grow without oxygen [8]. Both yeasts display the capacity to produce ethanol under aerobic conditions and the ability to tolerate high levels of ethanol, to grow under oxygen-limited conditions, and to survive without mitochondria [9–11]. These traits of *S*. *cerevisiae* are considered to be the consequence of yeast whole genome duplication (WGD) and promoter rewiring that happened approximately 100 million years ago [12–15]. It is apparent that the *Dekkera* yeasts did not undergo WGD. Nevertheless, *Dekkera* have been recognized as an alternative to *S*. *cerevisiae* in ethanol production [16, 17]. It is not clear how the *Dekkera* yeasts acquired the adaptive "winner" traits without WGD. The analysis of promoter sequences indicates that both *Dekkera* and *S*. *cerevisiae* independently underwent a massive loss of a specific *cis*-regulatory element in respiration genes, which contributes to the Crabtree effect [5]. However, the dysfunction of mitochondrial oxidative phosphorylation and the Crabtree effect may reinforce each other mutually. The causes and mechanisms involved in the Crabtree effect are not completely understood.

The decoding of the *Dekkera* genomes presented an opportunity to study the molecular evolution features in the *Dekkera* yeasts [8, 18]. In this study, *Dekkera bruxellensis* CBS 2499 and its nearest phylogenetic neighbor *Dekkera anomala* strain YV396, a species found to co-exist with *D*. *bruxellensis*, were chosen to represent the *Dekkera* yeasts [19]. We compared the *Dekkera* yeasts with five related non-WGD yeasts (*Y*. *lipolytica*, *K*. *lactis*, *L*. *kluyveri*, *L*. *waltii*, and *E*. *gossypii*) to investigate the independent evolution of "winner" traits in the *Dekkera* yeasts. The results indicated that the *Dekkera* yeasts and S. *cerevisiae* evolved independently to develop the "winner" traits with different evolutionary processes.

## Results

### Gene identification and annotation in the two *Dekkera* yeasts

**Fig 1:**
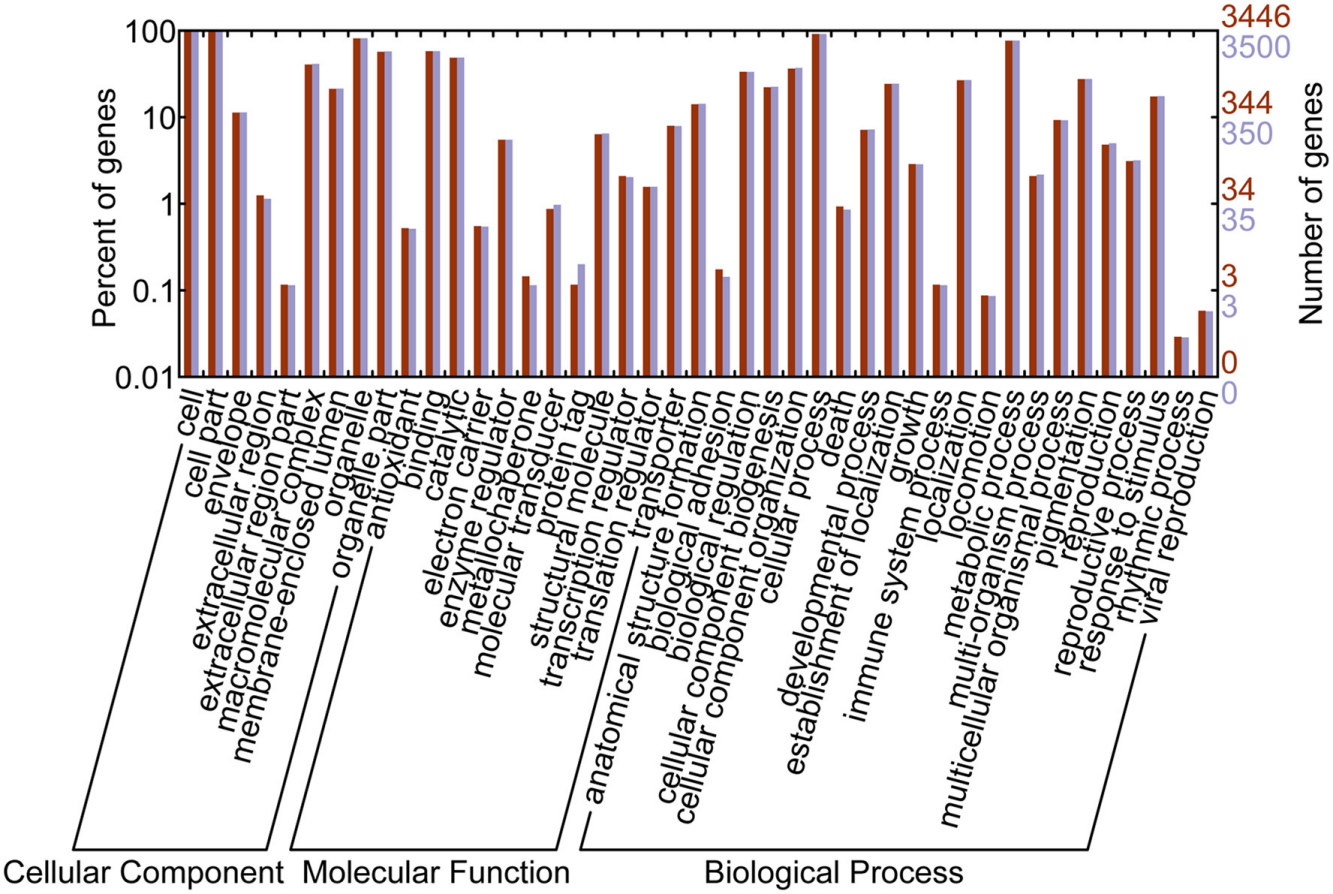
GO classification of annotated genes in *Dekkera bruxellensis* and *Dekkera anomala*. Three levels (Biological Process, Molecular Function, and Cellular Component) are indicated.

We used the AUGUSTUS online gene prediction tool to identify all of the possible protein coding open reading frames (ORFs) in the genomes of *Dekkera bruxellensis* CBS 2499 and *Dekkera anomala* strain YV396 [20]. In total, 5208 and 5241 ORFs were predicted for *D*. *bruxellensis* CBS 2499 and *D*. *anomala* YV396, respectively. Next, we utilized a reciprocal best-hits (RBH) BLAST approach for the prediction of ORFs in the *S*. *cerevisiae* protein-coding genes [21]. Ultimately, we annotated 3446 and 3500 genes which were one2one orthologous gene of *S*.*cerevisiae* in *D*. *bruxellensis* and *D*. *anomala*, respectively (S1 Table). All of these genes were used for further studies. Using WEGO, a web tool for plotting GO annotations, these annotated genes were mapped to 48 main GO categories in three domains (24 GO terms in Biological Process, 12 GO terms in Molecular Function, and 12 GO terms in Cellular Component) (Fig 1).

### The loss of Cis-elements in *D*. *bruxellensis* and *D*. *anomala* mitochondrial ribosomal genes

Modification of cis-elements at conserved sites is linked to the transcriptional network and regulation of gene expression, which have an intimate relationship with phenotypic diversity [13]. For example, the AATTTT motif, known as the rapid growth element (RGE), was found in the genes involved in rapid growth and respiration [22]. Previous studies have reported the profoundly massive loss of AATTTT motifs in the respiration-associated genes in *D*. *bruxellensis*, *S*. *cerevisiae*, and their sister species [5, 8]. To verify whether AATTTT motifs were also lost in *D*. *anomala* compared with the other yeasts, we collected three data sets: the CRP set (the rapid growth-associated genes, including the cytoplasmic ribosomal genes, ribosomal biogenesis and nuclear export of ribosomal subunits genes, and genes related to the purine biosynthesis pathway), the rRNA set (the rapid growth-associated genes, including the rRNA processing genes, genes coding for proteins involved in RNA biogenesis and transport, t-RNA transporters, pyrimidine biosynthesis pathway), and the MRP set (including mitochondrial ribosomal genes). We searched 600 bp of sequence upstream from these genes to identify promoter regions and analyzed the presence of the AATTTT motifs.

**Fig 2:**
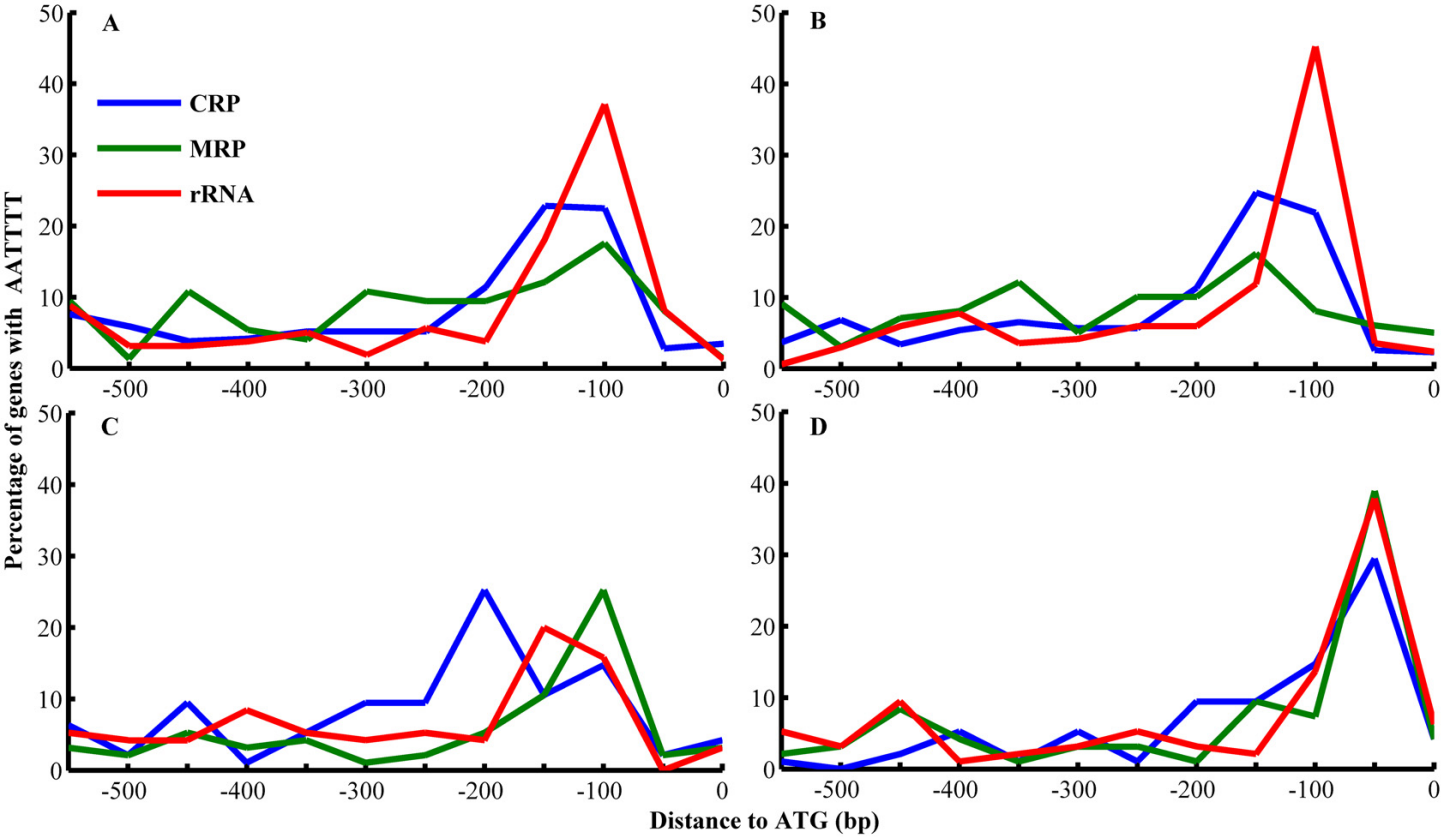
The MRP genes in the *D*. *bruxellensis* and *D*. *anomala* exhibited significant loss of the **AATTTT** motifs. The X-axis represents the distance to the start codon, and the Y-axis represents the percentage of genes with either the AATTTT motif or its reverse complement. The *blue*, *red*, and *green* lines represent CRP, rRNA, and MRP genes, respectively. A. *Dekkera bruxellensis*, B. *Dekkera anomala*, C. *Kluyveromyces lactis*, and D. *Lachancea waltii*.

As expected, the AATTTT motifs were enriched in the sequences which were 50–150 bp upstream from the start codon ATG. The patterns of AATTTT motifs in rRNA genes from *D*. *anomala* were nearly similar to those in *D*. *bruxellensis*. Approximately 40% of the rRNA genes possessed AATTTT motifs in the sequences 100 bp upstream of ATG. The AATTTT patterns of CRP genes and MRP genes in the *D*. *bruxellensis* and *D*. *anomala* (Fig 2A and 2B) were different from those in *K*. *lactis* and *L*. *waltii* (Fig 2C and 2D). The MRP and CRP genes of the *Dekkera* yeasts showed relatively lower percentages of AATTTT motifs 50–150 bp upstream of ATG compared with the rRNA genes. Nevertheless, the percentage of MRP genes in the *Dekkera* yeasts that contain an AATTTT motif was nearly half of that in *K*. *lactis and L*. *waltii* (15% versus 30%). This result illustrated that both the *Dekkera* yeasts lost *cis*-regulatory AATTTT motifs in the MRP gene promoters, similar to the post-WGD *S*. *cerevisiae*.

### Rapid evolution of coding sequences in the *D*. *bruxellensis* and *D*. *anomala*

**Fig 3:**
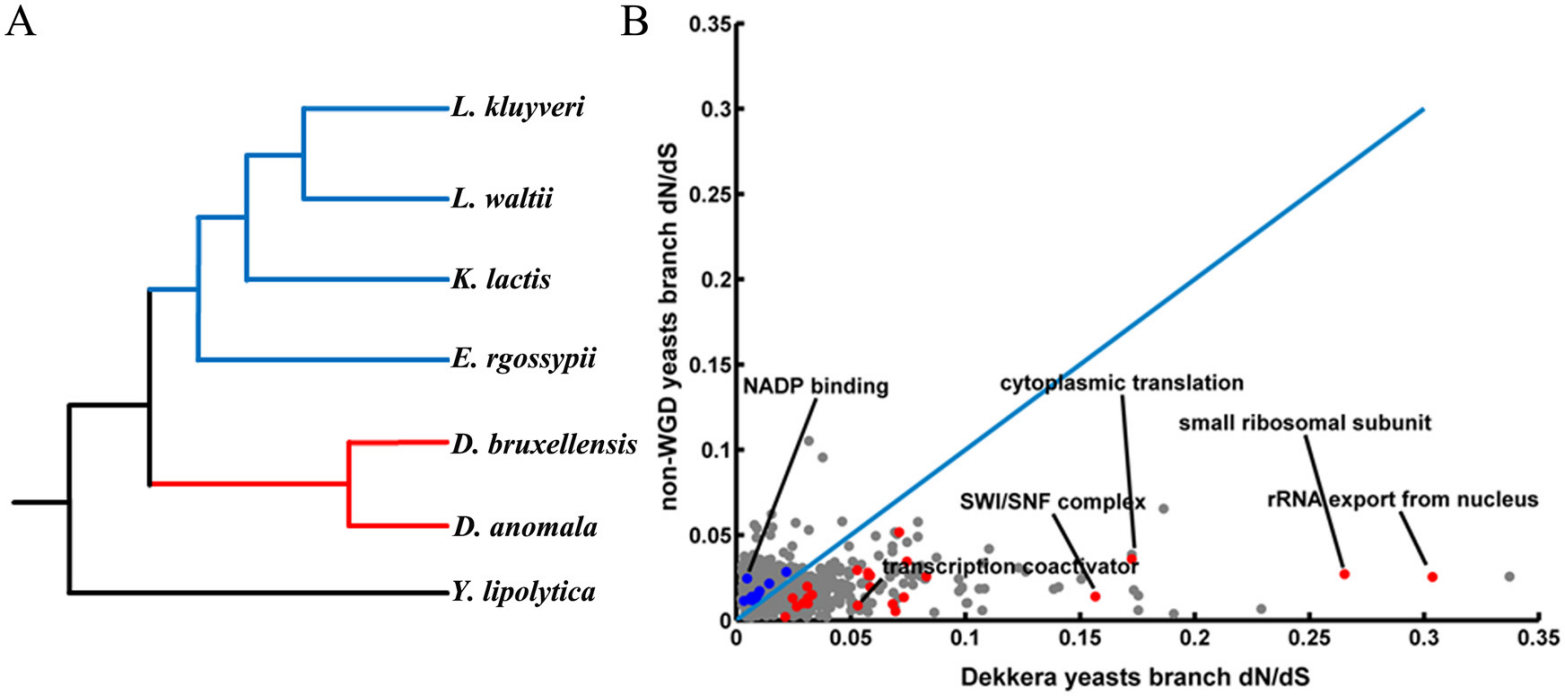
Evolution of coding genes in the *Dekkera* yeasts. (A) The phylogenetic tree of the *Dekkera* yeasts and five non-WGD yeasts. The *red* branches represent the *Dekkera* yeasts, and the *blue* branches represent the four non-WGD yeasts used for comparison. *Y*. *lipolytica* was the outlier group. (B) Comparison of dN/dS ratios between the *Dekkera* yeast branch and the other four non-WGD yeast branches by GO functional categories. The *red* and *blue* dots represent the categories with elevated evolutionary rates in the *Dekkera* yeast branch and the other yeast branches, respectively.

Analyses of orthologous genes suggested that rapid evolution occurred in the genes mediating the adaptation to produce high levels of ethanol and grow without oxygen. Among the 3500 predicted coding genes in the *Dekkera* yeasts, 2429 genes were annotated as high-confidence one-to-one orthologous genes among the seven non-WGD yeasts. These genes were used to estimate the evolutionary constraints acting on the *Dekkera* yeasts (Fig 3A). We calculated the dN/dS value for each GO term in the *Dekkera* branch and the other four non-WGD branches using the free ratio model implemented in PAML4 [23]. In total, 24 GO categories showed significantly higher dN/dS values in the *Dekkera* branch than those in the other branch yeasts (Adjusted P < 0.05, binomial test). These categories were primarily related to gene transcription and translation, including rRNA export from the nucleus (GO:0006407, P = 0.0031), small ribosomal subunit (GO:0015935, P = 0.039), *SWI/SNF* complex (GO:0031225, P = 0.009), cytoplasmic translation (GO:0002181, P = 0.0013), and transcription coactivator activity (GO:0003713, P = 0.013). A few categories showed lower dN/dS values in the *Dekkera* yeasts, such as NADP binding (GO:0050661, P = 0.021) (S2 Table, Fig 3B). The free ratio model also indicated that 748 (30.7%) genes evolved more than two-fold faster in the *Dekkera* yeasts, while 646 (26.5%) genes evolved faster in the other four non-WGD yeasts. A wide range of coding genes in the *Dekkera* yeasts experienced different evolutionary rates in these two branches, indicating that the *Dekkera* yeasts altered their physiological and metabolic processes, making them distinct from the other non-WGD yeasts.

To investigate the metabolic processes, we focused on the fermentation pathway related genes. The glycolysis pathway is the key metabolic pathway responsible for fermentation. At least one copy of the orthologous genes for the glycolytic pathway enzymes were detected among the seven yeasts examined. We used a branch model to distinguish the different rates of evolution in the *D*. *bruxellensis* and *D*. *anomala*, with the hypothesis that the *Dekkera* branch and the other non-WGD branches evolved at different rates. Our analysis identified 9 fermentation related genes that displayed different evolutionary rates between the *Dekkera* and other yeasts. Among them, *HXK2*, *LAT1*, *ACS2*, and *PDB1* underwent significantly slower evolution in the *Dekkera* yeasts. The other five genes, *ALD2*, *PYK1*, *PRM15*, *PGK1*, and *YOR283W* (*GPM1*-like protein) exhibited significantly faster evolution (S3 Table). Moreover, we found that the *PDC* gene in the *Dekkera* yeasts evolved into a form closer to the *PDC6* gene in *S*. *cerevisiae*. Transcription of *PDC6* is glucose- and ethanol-dependent and is strongly induced during sulfur limitation [24]. The Codon Adaptation Index (CAI) value of *PDC* in the *Dekkera* yeasts was significantly higher than the genome average level (0.46 and 0.31 versus 0.136 ± 0.062 and 0.139 ± 0.062 in *D*. *bruxellensis* and *D*. *anomala*, respectively), which suggests that *PDC* might be highly expressed in the *Dekkera* yeasts. As expected, under limited sugar supply and low concentrations of oxygen, the AB SOLiD sequencing technique showed that *PDC* is highly expressed in *Dekkera* (RPKM = 1330, rank 68) [25]. These results indicate that the *PDC6-*like gene may play a critical role in the adaptive evolution of the *Dekkera* yeasts.

### Positive selection in the *D*. *bruxellensis* and *D*. *anomala*

The branch-site model executed in PAML4 [23] identified 90 positively selected genes among the 2429 orthologous genes (3.7%) possibly associated with the adaptation of *Dekkera* yeasts (S4 Table). Using the MIPS and the SGD databases, we classified these genes into 10 functional categories (Table 1). These results demonstrated that the highest number of genes (53.9%) represented proteins with a binding function or cofactor requirement. Moreover, the metabolism and cell cycle DNA processing classes were over represented. Consistent with our rapid evolution study, 24 positive selection genes were mapped into the transcription functional class. For example, *CLU1*, *NIP1*, and *SUI2* were subunits of translation initiation factors; and *TCF1* and *TFC3* were subunits of the RNA polymerase III transcription initiation factor complex. This suggested that although the *Dekkera* yeasts have a single copy of the genome, they may have acquired the "winner" traits by evolving an efficient translation system to improve protein levels. This could explain why glycolysis genes are expressed at high levels in the *Dekkera* yeasts. In addition, we found that 12 genes were represented in vacuolar function, which is required for tolerance to alcohol stress [26]. Genes, such as *END3*, *CHC1*, and *PEP3*, are required for vacuolar biogenesis; *MNN2*, which encodes mannosyltransferases, is involved in maintaining cell wall integrity. One of the three rate-limiting enzymes in glycolysis, 3-Phosphoglycerate Kinase (*PGK1*), was found to be under positive selection. *PGK1* catalyzes the reversible transfer of high-energy phosphoryl groups from the acyl phosphate of 1,3-bisphosphoglycerate (1,3-BPG) to ADP, producing 3- phosphoglycerate (3-PG) and ATP. The catalytic rate of the *PGK1* enzyme determines the flux of carbon degradation in the direction of pyruvate or glycerol. Because efficient anaerobic fermentation needs a higher glycolysis flux (GF), we speculate that the positive selection sites in these two glycolysis rate limiting enzymes represent adaptive evolution to improve enzyme activities in the *Dekkera* yeasts.

**Table 1:** Classification of genes under positive selection in the *D*. *bruxellensis* and *D*. *anomala*.

**Metabolism (28)**	*PTC7*, *BIG1*, *RKM3*, *MEC1*, *FAT1*, *YBR056W*, *CYC8*, *MNN2*, *TDP1*, *PGK1*, *PHO2*, *SSN2*, *SSY1*, *PPM1*, *EDC3*, *YER134C*, *BST1*, *MSB2*, *STR2*, *SWI3*, *TRZ1*, *SET3*, *RIX7*, *CAR2*, *RNH1*, *CDC5*, *DSS1*, *ORT1*
**Transcription (24)**	*TFC3*, *CYC8*, *TFC1*, *PIM1*, *PHO2*, *PRP42*, *SSN2*, *SSY1*, *MSS2*, *PCF11*, *HAT2*, *VHR1*, *TAO3*, *STS1*, *SWI3*, *PRI2*, *TRZ1*, *SET3*, *DSS1*, *LEO1*, *CLP1*, *CTR9*, *HDA3*, *NAB3*
**Vacuolar function (12)**	*BST1*, *END3*, *SEC16*, *CHC1*, *COG6*, *SEC10*, *SEC15*, *GYP5*, *LTE1*, *PEP3*, *NYV1*, *BRO1*
**Cell cycle and DNA processing (31)**	*SPC97*, *BIG1*, *LTE1*, *MEC1*, *KAP104*, *TDP1*, *MRC1*, *CDC1*, *CYK3*, *HAT2*, *BEM2*, *SEC15*, *CUL3*, *STS1*, *DPB11*, *RAD7*, *SWI3*, *PRI2*, *SET3*, *RIX7*, *SEC10*, *BUD6*, *RNH1*, *CDC5*, *CLU1*, *NDC1*, *DSS1*, *END3*, *CTR9*, *HDA3*, *GYP5*
**Protein with binding function or cofactor requirement (48)**	*LTE1*, *MEC1*, *FAT1*, *UMP1*, *KAP104*, *PBY1*, *CYC8*, *PIM1*, *PGK1*, *PRP42*, *SSY1*, *MSN5*, *PCF11*, *HAT2*, *BEM2*, *YER134C*, *SEC15*, *CHC1*, *CUL3*, *TAO3*, *SUI2*, *RAD7*, *SWI3*, *PRI2*, *TRZ1*, *SET3*, *RPL38*, *PEP3*, *SPA2*, *RIX7*, *CAR2*, *CRN1*, *SEC10*, *BUD6*, *MVP1*, *RNH1*, *CDC5*, *DSS1*, *NIP1*, *END3*, *MTR10*, *CLP1*, *CTR9*, *BRO1*, *GYP5*, *SRP72*, *NAB3*, *SEC16*
**Regulation of metabolism and protein function (14)**	*PTC7*, *LTE1*, *MEC1*, *UMP1*, *CYC8*, *HAT2*, *BEM2*, *YER134C*, *STE2*, *SUI2*, *CDC5*, *WHI2*, *BRO1*, *GYP5*
**Cell rescue, defense and virulence (13)**	*MEC1*, *UMP1*, *TDP1*, *PIM1*, *PHO2*, *MSB2*, *SUI2*, *RAD7*, *PRI2*, *BUD6*, *DSS1*, *WHI2*, *BRO1*
**Interaction with the environment (13)**	*PIM1*, *CDC1*, *SSY1*, *MSN5*, *HAT2*, *STE2*, *MSB2*, *SYG1*, *SWI3*, *SPA2*, *END3*, *CTR9*, *BRO1*
**Biogenesis of cellular components (25)**	*SPC97*, *BIG1*, *MEC1*, *TFC1*, *MNN2*, *CDC1*, *HAT2*, *BEM2*, *BST1*, *SEC15*, *MSB2*, *CHC1*, *SWI3*, *SET3*, *PEP3*, *SPA2*, *RIX7*, *CRN1*, *BUD6*, *RNH1*, *CLU1*, *NDC1*, *END3*, *WHI2*, *GYP5*
**Energy (4)**	*FAT1*, *PGK1*, *MSB2*, *RIX7*

**Fig 4:**
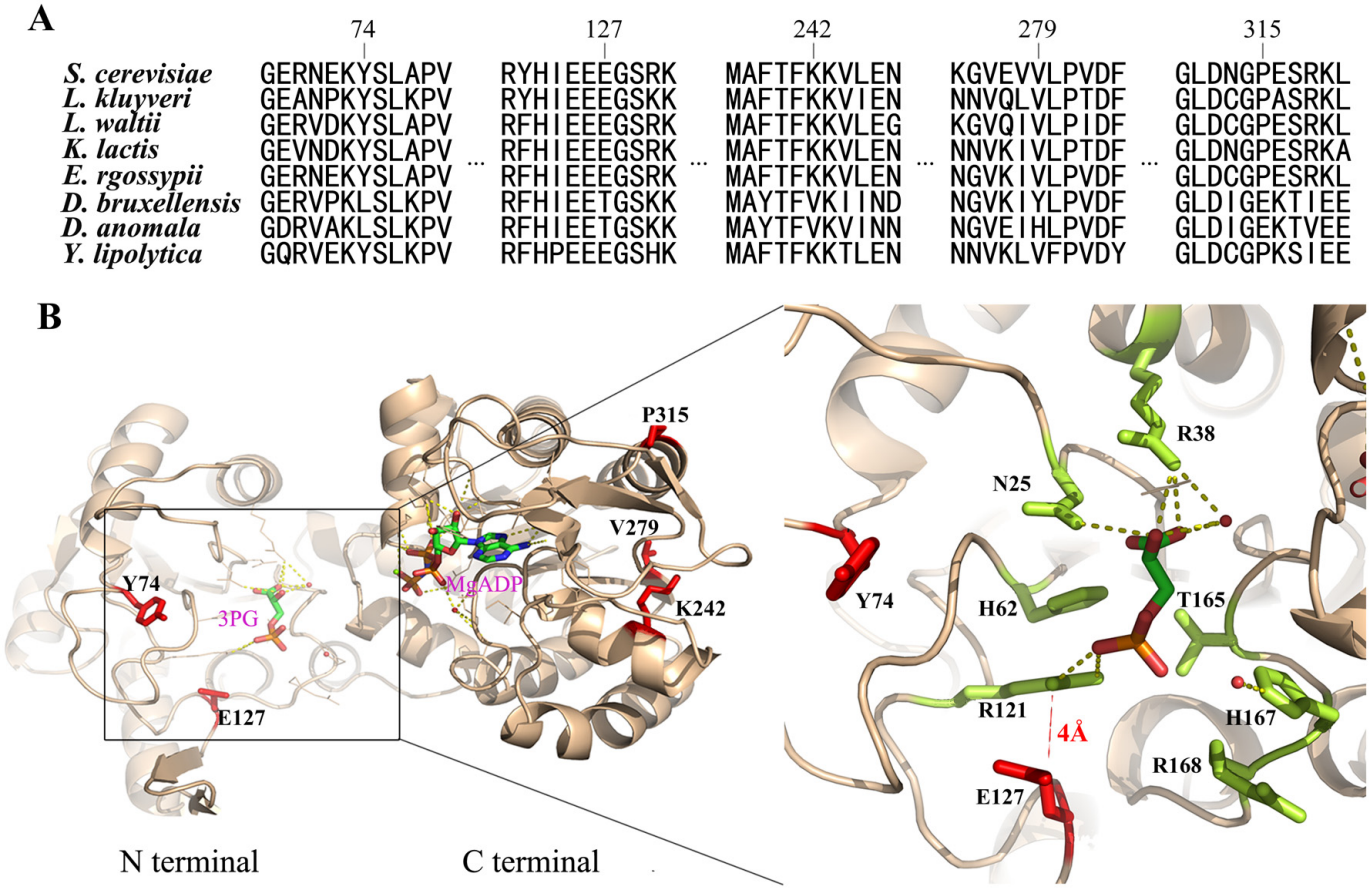
Positive selection sites in *D*. *bruxellensis* and *D*. *anomala PGK1*. (A) Sequence alignment of *PGK1* in all 8 species. (B) The three-dimensional structure of *PGK1* in *S*. *cerevisiae*. Left panel: The selection sites in the *Dekkera* yeasts are highlighted in *red*. The *pink* 3PG and MgADP represent binding conformations of the two substrates, respectively. Right panel: Details of the 3PG binding pocket. The distance between E127 and R121 is shown by the *red dashed line*.

Aligned amino acid sequences of the seven yeast species showed that the Y74L, E127T, K242V, V279Y, and P315E mutations are simultaneously retained in the *Dekkera PGK1* (Fig 4A). Then, we analyzed the three-dimensional structure of *S*. *cerevisiae PGK1* to predict the impact of selective mutations on the rate of enzyme catalysis (Fig 4B). Yeast *PGK1* exists as a 415-residue monomer containing two nearly equal-sized domains that correspond to the N- and C-termini of the protein [27]. 3-PG and 1,3-BPG bind to the N-terminal domain, while the nucleotide substrates, MgATP or MgADP, bind to the C-terminal domain of the enzyme. This extended two-domain structure is associated with large-scale "hinge-bending" conformational changes, similar to those found in hexokinase [28]. Binding of either substrate triggers a conformational change, and the transfer of the phosphate group occurs when both substrates are bound [29]. *PGK1* catalyzes the glycolytic direction in the glycolysis pathway and catalyzes its reverse direction in the gluconeogenic pathway. In our study, the five positive selection sites were found to be located at both N- and C-termini of *PGK1*. The three C-terminal selective sites, located in the region surrounding the 6-stranded parallel beta-sheet and alpha helices, may help the C-terminal domain fold into a specific conformation. Y74L and E127T, which are located in the N-terminus, are situated very close to the binding pocket of 3PG and 1,3-BPG. Particularly, the negatively charged side chain of the E127 residue was at a distance of merely 4 Å to the positively charged side chain of R121 in *S*. *cerevisiae*. The R121 residue plays a key role in the stabilization of the phosphate groups of 3-PG and 1,3-BPG. However, in the *D*. *bruxellensis* and *D*. *anomala*, as the negatively charged E127 residue is mutated to a non-charged T127, the influence of reverse charge for R121 would disappear. Therefore, the electrostatic interaction between R121 and the phosphate group of 3PG would be enhanced. However, as 1,3-BPG has two phosphate groups and the R121 of *PGK1* enhances the non-reaction phosphate group, the reaction group would be pulled down from 1,3-BPG more easily. Thus, the catalytic activity of *PGK1* would be redirected to the glycolytic pathway in the *Dekkera* yeasts as a consequence of positive selection.

## Discussion

The *Dekkera* yeasts represent an alternative to ethanol production using *S*. *cerevisiae*. The *Dekkera* yeasts independently evolved "winner" traits, such as high glucose and ethanol tolerance, aerobic fermentation, and rapid ethanol fermentation rate, similar to *S*. *cerevisiae*. Previous phylogenetic analysis suggests that the *S*. *cerevisiae* and *Dekkera* groups separated at least 200 Mya, which took place long before the WGD in *S*. *cerevisiae*. Promoter rewiring, *URA1* horizontal transfer, and *ADH* duplication events are thought to be involved in the "make-accumulate-consume" strategy in *S*. *cerevisiae* [9, 15]. The WGD event provided the *S*. *cerevisiae* with extremely rich evolutionary resources for adapting to the pressure of high levels of sugars 100 mya. The *Dekkera* yeasts independently accomplished this adaptation without a duplicated genome, and often occupy a similar niche to the *S*. *cerevisiae* yeasts in nature.

In this study, we analyzed the genomes of two *Dekkera* yeasts: *Dekkera bruxellensis* and *Dekkera anomala*. By comparison with five closely related non-WGD yeasts, we revealed that the AATTTT motifs of MRP genes are lost on a large scale not only in *D*. *bruxellensis*, which was consistent with previous studies, but also in *D*. *anomala*. This result indicated that the loss of the AATTTT motif occurred in a common ancestor of *D*. *bruxellensis* and *D*. *anomala*. However, the global promoter rewiring can only explain the change in the expression pattern of respiration-associated genes. How did *Dekkera* adapt to the high levels of glucose and ethanol and produce ethanol efficiently? In a quest to unravel the potential genetic mechanisms underlying these adaptations, we found, surprisingly, that a multitude of coding genes showed significantly different evolutionary rates in the *Dekkera* yeasts compared with the other non-WGD yeasts. GO enrichment analysis showed that genes involved in the transcription and translation processes exhibited faster rates of evolution in the *Dekkera* yeasts. In addition, approximately 3.7% of coding genes contained at least one positively selected amino acid in the *D*. *bruxellensis* and *D*. *anomala*. Positive selection of *CLU1*, *NIP1*, *SUI2*, *TCF1*, and *TFC3* indicated that the *Dekkera* yeasts evolved a very efficient transcription and translation system. Positively selected genes in the cell rescue, defense and virulence, and vacuolar function classes provide a reasonable explanation for the tolerance of the *Dekkera* yeasts to high levels of alcohol and glucose. For example, the *SPC97*, *MNN2*, *CHC1*, *NYV1*, and *MVP1* genes, which are important for vacuolar H^+^-ATPase function, may play a key role in high alcohol tolerance. Moreover, more than half of the positively selected genes were found to encode enzymes related to metabolism. These findings indicated that the metabolic process in the *Dekkera* yeasts acquired several new traits through adaptive evolution.

Tolerance to high levels of glucose and ethanol and the efficient rate of ethanol production point to the adaptive evolution of the fermentation pathway in the *Dekkera* yeasts. Positively selected and differentially evolved genes in the fermentation pathway may explain, in part, the molecular genetic mechanism for adaptive evolution. The rapid rate of ethanol production needs a relatively higher fermentation flux. Previous studies illustrated that the *Dekkera* yeasts express a high number of sugar transporter genes, indicating a higher rate of sugar uptake [25]. In our study, we suggest that glycolysis for sugar degradation would be accelerated in the *Dekkera* yeasts as a consequence of positive selection in *PGK1*. We surmise that the selection sites in *PGK1* make pyruvate the preferred product of sugar degradation compared with glycerin. After analysis of the crystal structure of *PGK1*, we found that the positive selection site E127T in *PGK1* enhanced its catalytic activity in the glycolytic direction in preference to gluconeogenesis. In addition, we found that the *PDC* gene in the *Dekkera* yeasts is unique and closer to *PDC6* in *S*. *cerevisiae*. In contrast, PDC in the other non-WGD yeasts was more akin to *PDC1*. Expression of *PDC6* is induced by glucose in conditions of high glucose. The *Dekkera* yeasts express high levels of this unique *PDC6-*like protein to convert pyruvate to acetaldehyde rapidly. Former studies also indicated that the *ADH* genes are duplicated in both *Dekkera* and *S*. *cerevisiae*, so *Dekkera* can easily convert acetaldehyde to ethanol [8]. These genetic features illuminate the evolution of a high efficiency sugar transport and degradation system in the *Dekkera* yeasts to produce ethanol rapidly. When glucose is depleted and ethanol levels are high, another gene that underwent fast evolution, *ALD2*, would be highly expressed in *Dekkera* and convert acetaldehyde to the acetic acid. The acetic acid could convert into acetyl-CoA by *ACS*, and then acetyl-CoA could be used in the TCA cycle in aerobic respiration. Moreover, the *GRR1* gene and the *TDH2-*like gene may also be helpful for the adaptation to grow in high levels of glucose (S1 Table) [30–32].

In conclusion, by analyzing the genome of *D*. *bruxellensis* and *D*. *anomala* and five non-WGD yeasts, we uncovered some molecular bases for the "winner" traits in the *Dekkera* yeasts, which may explain the adaptive evolution of *Dekkera*. Some mutations in *Dekkera* may have contributed to the increased metabolic capacity to degrade sugar to ethanol. These findings may be useful in an industrial setting. For example, modification of *PGK1* can be used to improve ethanol production by bio-engineering *PGK1* in *S*. *cerevisiae*. Thus, our study unraveled some of the molecular mechanisms underlying the adaption evolution of the *Dekkera* yeasts and provides a blueprint for genetic modification for industrial usage.

## Materials and Methods

### 1. Acquisition and annotation of the sequence data

The genomes of *Yarrowia lipolytica*, *Kluyveromyces lactis*, *Lachancea kluyveri*, and *Lachancea waltii* were obtained from the Yeast Gene Order Browser (http://ygob.ucd.ie/) [33]. The genomes of *Dekkera bruxellensis* CBS 2499 and *Dekkera anomala* strain YV396 were downloaded from the NCBI genome database (http://www.ncbi.nlm.nih.gov/genome/). The genome of *Eremothecium gossypii* was downloaded from the Yeast Genomes database [34] (http://www.genolevures.org/). We downloaded the detailed latest release version sequence and annotation information of *Saccharomyces cerevisiae* (S288C), as a reference to annotate known genes in the sequenced species, from the Saccharomyces Genome Database (http://yeastgenome.org/) [35]. We utilized the AUGUSTUS program to predict ORFs to annotate and identify orthologous genes between *Saccharomyces cerevisiae* and the other yeasts. Then, a reciprocal best-hits (RBH) BLAST approach and the inparanoid4.1 software package were used to detect orthologs between any two yeasts [36]. We removed all reduplicated genes from the analysis, for example one orthologous cluster containing more than one ortholog [33]. We retained orthologous clusters that had only one gene from each species.

### 2. Cis-element regulator motif analysis

The genes included in this study were divided into three sets: the CRP and rRNA sets, associated with the rapid growth-associated genes, and the MRP set, associated with the mitochondrial ribosomal genes. We used the proposed datasets of *Saccharomyces cerevisiae* (the CRP, rRNA, and MRP sets contain 174, 61 and 72 genes, respectively). Genes from the three sets were mapped to the genomes of the four yeast species (all of the sequenced DNA databases were annotated using *S*. *cerevisiae*) to extract genes belonging to the three sets from the different yeast genomes. We defined promoter sequences by searching sequences 600 bp upstream of the genes in every set. 600 bp of the promoter sequences were divided into 12 ordered groups (according to the position of nucleotides) and the presence of AATTTT motifs and reverse complements were identified separately. All promoter sequences belonging to the same set were counted as the total number of motifs present in each ordered 50 bp window. Then, we calculated the percentage of genes with AATTTT motifs in each 50 bp window and portrayed a broken line graph. We used python scripts developed in-house to define the CRP, rRNA, and MRP sets and analyze the occurrence of regulatory AATTTT motifs. The significance of motif distribution between the four species was detected using the repeated-measures analysis of variance.

### 3. Calculation of evolutionary rate

We used the dN/dS ratio to measure the evolutionary rate along a lineage. The values of dN, dS, and the dN/dS ratio were calculated using the free ratio model (Parameters of one rate model: model = 1, NSsites = 0, fix_omega = 0, omega = 1) in PAML4 for the *Dekkera* branch and the other branches. Lineage-specific median values were estimated by concatenated alignments from all orthologs. GO term data were downloaded from the Gene Ontology Consortium [37], and GO categories with more than 5 orthologs were included in our analyses. GO terms experiencing relatively accelerated evolution were identified using a binomial test [38]. The evolution rate for the glycolysis pathway was estimated using the branch model (Parameters of one rate model, background: model = 0, NSsites = 0, fix_omega = 0, omega = 0.2; Different rate model, foreground: model = 2, NSsites = 0, fix_omega = 0, omega = 0.2). The accepted species-tree (*Y*. *lipolytica*, ((*E*. *gossypii*, (*K*. *lactis*, (*L*. *kluyveri*, *L*. *waltii*))), (*D*. *bruxellensis* #1, *D*. *anomala* #1) #1)) was generated using the MEGA6 program [39] and was used as the guide tree. Ultimately, the likelihood ratio test (LRT) was performed to find genes with remarkable difference between the foreground and background. Level of significance was set at P< 0.05, i.e., the value of chi-square is equal to or greater than 3.84. Amino acid sequences were aligned using ClustalW2 [40] and the pal2nal [41] software was used to match the nucleotide sequences.

### 4. Identification of positively selected genes

We calculated the ratio of nonsynonymous (Ka or dN) to synonymous (Ks or dS) substitution rate (ω = Ka/Ks or dN/dS) using CODEML in the PAML4 package. Using the guide tree, the branch-site model (Parameters: Null hypothesis: model = 2, NSsites = 2, fix_omega = 1, omega = 1; Alternative selective hypothesis: model = 2, NSsites = 2, fix_omega = 0, omega = 1) was used to detect positively selected genes. A likelihood ratio test compared the alternative hypothesis of positive selection on the foreground branch to a null hypothesis with no positive selection on the background branch for each orthologous gene. Positively selected genes were inferred only if their P values were less than 0.05. Positively selected sites were deduced using Bayes Empirical Bayes (BEB) analyses [42]. Specifically, we edited the aligned amino acid sequences using the BioEdit software and drew protein conformation using the PyMOL software. The structure of PGK was downloaded from the protein data base (PDB) with PDB id: 1QPG.

## Supporting Information

S1 Table Predicted one2one orthologous gene of *S*. *cerevisiae* genes in 7 non-WGD species. (XLS)

S2 Table The evolutionary rate of GO categories between the *Dekkera* and other non-WGD yeasts. (XLS)

S3 Table Genes with significantly different evolution rates in the fermentation pathway related genes. (XLS)

S4 Table Positive selection sites in the *Dekkera* yeasts. (XLS)
